# Mining TCGA Database for Tumor Microenvironment-Related Genes of Prognostic Value in Hepatocellular Carcinoma

**DOI:** 10.1155/2019/2408348

**Published:** 2019-11-19

**Authors:** Zhenfeng Deng, Jilong Wang, Banghao Xu, Zongrui Jin, Guolin Wu, Jingjing Zeng, Minhao Peng, Ya Guo, Zhang Wen

**Affiliations:** ^1^Department of Hepatobiliary Surgery, The First Affiliated Hospital of Guangxi Medical University, Nanning 530021, China; ^2^Department of Pathology, The First Affiliated Hospital of Guangxi Medical University, Nanning 530021, China

## Abstract

Hepatocellular carcinoma (HCC) is one of the most common and lethal malignancies. Recent studies reveal that tumor microenvironment (TME) components significantly affect HCC growth and progression, particularly the infiltrating stromal and immune cells. Thus, mining of TME-related biomarkers is crucial to improve the survival of patients with HCC. Public access of The Cancer Genome Atlas (TCGA) database allows convenient performance of gene expression-based analysis of big data, which contributes to the exploration of potential association between genes and prognosis of a variety of malignancies, including HCC. The “Estimation of STromal and Immune cells in MAlignant Tumors using Expression data” algorithm renders the quantification of the stromal and immune components in TME possible by calculating the stromal and immune scores. Differentially expressed genes (DEGs) were screened by dividing the HCC cohort of TCGA database into high- and low-score groups according to stromal and immune scores. Further analyses of functional enrichment and protein-protein interaction networks show that the DEGs are mainly involved in immune response, cell adhesion, and extracellular matrix. Finally, seven DEGs have significant association with HCC poor outcomes. These genes contain *FABP3*, *GALNT5*, *GPR84*, *ITGB6*, *MYEOV*, *PLEKHS1*, and *STRA6* and may be candidate biomarkers for HCC prognosis.

## 1. Introduction

Hepatocellular carcinoma (HCC) is one of the most common and deadly malignancies worldwide, with approximately 841,000 new cases and 782,000 deaths annually [1]. Currently, the treatment strategies of HCC include surgical resection, transplantation, radiofrequency ablation, transarterial chemoembolization, chemotherapy, and radiotherapy [2]. However, the effectiveness of these therapies is limited in most patients with HCC diagnosed at the middle or advanced stages. A latest study shows that high genetic heterogeneity of HCC may be the main cause of treatment failure [3], and biological and clinical diversities of HCC present great challenges in individualized clinical treatment [4–6]. Genomic heterogeneity in tumor cells has been widely investigated to identify different prognoses and therapeutic responses in subgroups of patients with HCC and to find new molecular targets [7–9]. Moreover, accumulating evidence indicates that nontumor cells in tumor microenvironment (TME) significantly influence the gene expression of tumor cells, which subsequently affects clinical outcomes [9–12]. TME is the microenvironment where the tumor cells are located; other than tumor cells, TME also consists of immune cells, fibroblasts, endothelial cells, extracellular matrix, cytokines, chemokines, and receptors [13]. Stromal and immune cells are two main types of nontumor components in the TME, and the investigation of their interaction has been valuable for developing innovative HCC-directed immunotherapies [14]. However, most previous studies about the HCC TME focused on immune microenvironment and the landscape of stromal cells in the TME lacks in-depth research. Recently, an algorithm that uses gene expression signatures to infer the fraction of stromal and immune cells and predict tumor purity in tumor samples has been developed. The algorithm is described as “Estimation of STromal and Immune cells in MAlignant Tumors using Expression data” (ESTIMATE) [15], which can help to understand the landscape of stromal and immune cells in the TME. Some reports have applied the ESTIMATE to colon cancers [16] and glioblastoma [17], revealing the effectiveness of such big-data-based algorithms. However, the effectiveness of stromal and immune scores in HCC has not been elaborated. In this study, we first calculated the stromal and immune scores of HCC cohorts from The Cancer Genome Atlas (TCGA) database by applying the ESTIMATE algorithm and extracted a list of TME-related genes that predict poor outcomes in patients with HCC.

## 2. Materials and Methods

### 2.1. Data Source

Publicly available dataset of HCC cohort, including Level 3 data of gene expression profile and relevant clinical information, was downloaded from TCGA data portal (https://portal.gdc.cancer.gov/, accessed May 21, 2019). The clinical information includes age, gender, liver fibrosis/cirrhosis status, pathologic stage, histologic grade, values of serum alpha fetoprotein (AFP), Child-Pugh score, microvascular invasion (MVI), radical resection, and survival time. The stromal and immune scores were calculated by applying the ESTIMATE algorithm to the downloaded RNA expression data, and HCC cases were categorized in accordance with the median of immune/stromal scores into high- and low-score groups. All data involved in this study were downloaded from TCGA, and data acquirement and application were performed in accordance with TCGA publication guidelines and data access policies. Thus, additional approval by the local Ethics Committee was not needed.

### 2.2. Calculation of Stromal and Immune Scores

The stromal and immune scores were calculated by applying the ESTIMATE algorithm to the downloaded RNA expression data, and HCC cases were categorized in accordance with the median of immune/stromal scores into high- and low-score groups. ESTIMATE outputs stromal and immune scores by performing single-sample gene set-enrichment analysis [15, 18]. For tumor samples of HCC cohort, first, gene expression values were rank-normalized and rank-ordered. Then, the empirical cumulative distribution functions were calculated for genes in the signature and the remaining genes. Finally, a statistic was calculated by an integration of the difference between the empirical cumulative distribution function.

### 2.3. Construction of Prognostic Signature Based on Stromal and Immune Scores

To explore the relevant contribution of stromal and immune scores to HCC survival prediction, they were fitted into a multivariate Cox regression analysis with survival time as the dependent variable. A prognostic risk score model was performed by the linear combination of the stromal and immune scores with the multivariate Cox regression coefficient (*β*) as the weight. The risk score formula was as follows: risk score = stromal score × *β*1 + immune score × *β*2 [19]. This prognostic model could divide the HCC cohort into high- and low-risk groups using the median risk score that was based on stromal and immune scores. The time-dependent receiver operating characteristic (ROC) curve was conducted using the “survivalROC” package (version 1.0.3) on the R platform to evaluate the predictive accuracy of this prognostic risk score model [20].

### 2.4. DEG Screening

Data analysis was performed by using the “limma” package (version 3.40.2) [21] on the R (version 3.6.0). Genes with a mean value >0 were included in the screening of DEGs. False discovery rate (FDR) <0.05 and |log2 fold change (log2FC)| ≥ 1.5 were set as the cut-offs to screen for DEGs.

### 2.5. Functional and Pathway Enrichment Analyses

The DEGs were analyzed by using the “clusterProfiler” R package (version 3.12.0) [22] for Gene Ontology (GO) terms and Kyoto Encyclopedia of Genes and Genomes (KEGG) database pathways. The GO analysis reveals the DEG function in biology process, cell component, and molecular function, and the KEGG analysis shows the pathway enrichment of DEGs. The adjusted *P* value < 0.05 was considered statistically significant.

### 2.6. Construction of Protein-Protein Interaction (PPI) Network

The PPI network of DEGs was retrieved through the Search Tool for the Retrieval of Interacting Genes (STRING, https://string-db.org/) [23] and reconstructed via Cytoscape software (version 3.7.1) [24], which is an open-source software platform for visualizing complex networks and integrating these with any type of attribute data. The Molecular Complex Detection plugin of Cytoscape was then used to find the most significant module based on topology to locate densely connected regions. The settings of selection were as follows: degree cut-off = 2, node score cut-off = 0.2, k-core = 2, and maximum depth = 100.

### 2.7. Statistical Analysis

Kaplan–Meier survival analysis by log-rank test was used to identify the TME-related DEGs regarding HCC poor prognosis. Univariate analyses between clinical characteristics and stromal/immune scores were compared using the log-rank test. A value of *P* < 0.05 was considered statistically significant. Venn diagrams, heat maps, and survival curves were plotted by R platform. Statistical analysis was performed using SPSS 22.0 (Chicago, IL, USA).

## 3. Results

### 3.1. Study Population and Their Stromal and Immune Scores

**Figure 1:**
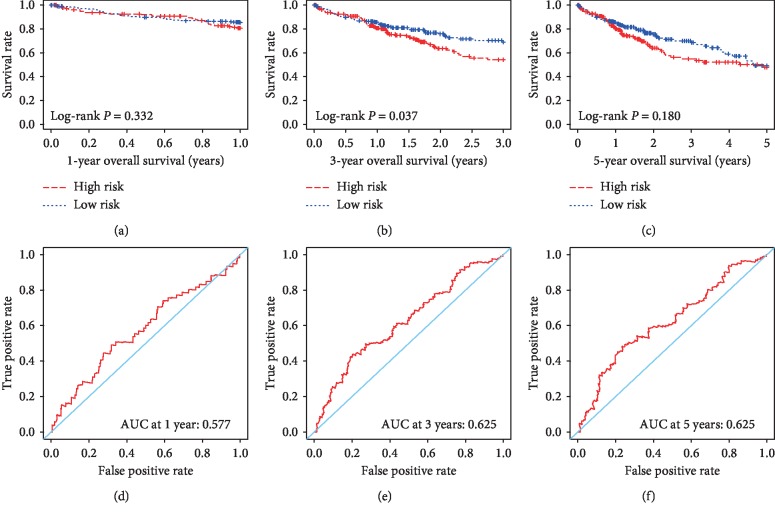
The Kaplan–Meier and ROC curves of prognostic risk score model based on stromal and immune scores in HCC. Kaplan–Meier curves of high- and low-risk groups for 1-year OS (a), 3-year OS (b), and 5-year OS (c); ROC curves of risk score model for 1-year OS (d), 3-year OS (e), and 5-year OS (f).

A total of 374 cases were available from the TCGA database for further analysis. Their RNA expression data of tumor tissues were used to calculate the stromal and immune scores. Based on the ESTIMATE algorithm, the range of stromal score was from −1,625.38 to 1,171.12, and the range of immune score was from −866.31 to 3,146.06. The relevant clinical data were also downloaded to investigate correlation with stromal and immune scores (Table 1). Univariate analysis identified the following clinical features as significantly associated with stromal scores: histologic grade (log-rank *P*=0.014), serum AFP (log-rank *P*=0.013), and MVI (log-rank *P*=0.018), and liver fibrosis/cirrhosis status was also significantly associated with immune scores (log-rank *P*=0.013). To explore the potential correlation of overall survival (OS) with stromal and immune scores, 374 HCC cases were divided into high- and low-score groups in accordance with the stromal/immune scores. Survival analysis shows that the median survival time (MST) of cases in the low-score group of stromal scores is longer than that in the high-score group (MST: 453 vs. 631 days; log-rank *P*=0.256). Cases with lower immune scores also showed longer MST compared with cases with higher immune scores (MST: 500 vs. 602 days; log-rank *P*=0.377), but both were not statistically significant at the *P* < 0.05 level and relevant figures are not shown. A multivariate Cox regression analysis was applied to further assess the relative contribution of the stromal and immune scores in survival prediction. The risk score formula was as follows: risk score = stromal score × (−0.1074) + immune score × (−0.4074). Survival analysis shows that patients with a high-risk score have a shorter MST than those with a low-risk score in 1-year OS (MST: 299 vs. 311 days; log-rank *P*=0.332; Figure 1(a)), 3-year OS (MST: 545 vs. 633 days; log-rank *P*=0.037; Figure 1(b)), and 5-year OS (MST: 666 vs. 763 days; log-rank *P*=0.180; Figure 1(c)). The area under the curve (AUC) of ROC curve was 0.577, 0.625, and 0.625 for 1-, 3-, and 5-year survival based on the time-dependent ROC analysis (Figures 1(d)–1(f)).

### 3.2. DEG Screening

**Figure 2:**
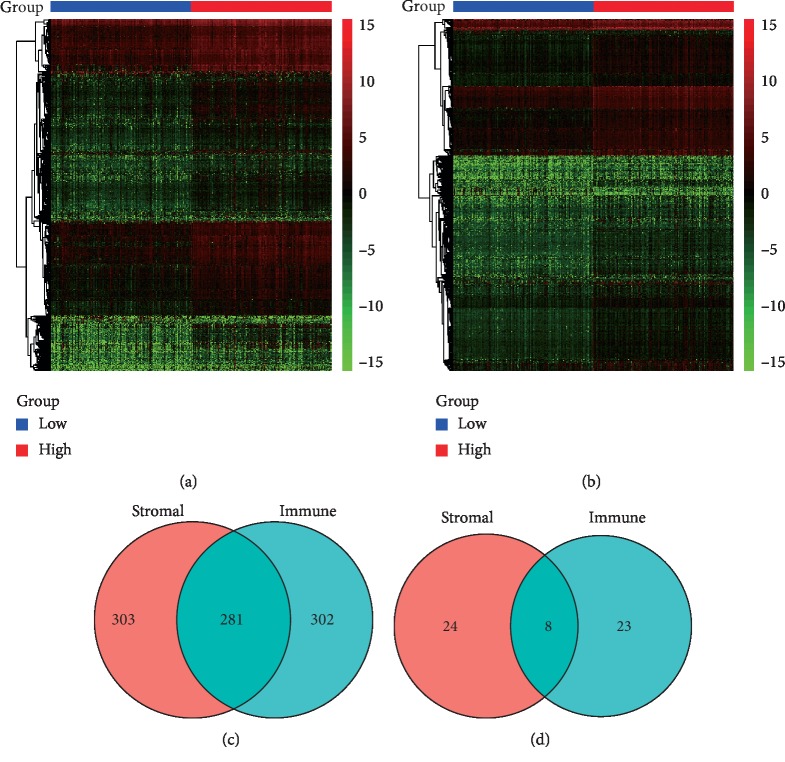
Comparison of gene expression profile with stromal and immune scores in HCC. Heat map of DEGs between high and low groups in (a) stromal scores and (b) immune scores. In drawn heat maps, genes with higher expression are shown in red, while lower expressions are shown in green, and the same expression levels are in black. Venn diagrams show the number of (c) co-upregulated or (d) co-downregulated genes between stromal and immune groups.

Unique gene expression profiles of 374 cases were shown in heat maps by categorizing the HCC cohort into high- and low-scores groups of stromal/immune cells (Figures 2(a) and 2(b)). For comparing high and low groups based on the stromal scores, 584 upregulated genes and 32 downregulated genes were identified. For the immune score groups, 583 upregulated genes and 31 downregulated genes were identified. In addition, Venn diagrams showed that 281 identical genes were upregulated and 8 identical genes were downregulated between the stromal and immune score groups (Figures 2(c) and 2(d)). Therefore, a total of 289 genes were screened as DEGs after taking the intersection by drawing Venn diagrams, which meets the criteria of FDR <0.05 and |log2FC| ≥ 1.5.

### 3.3. Functional Assessment

**Figure 3:**
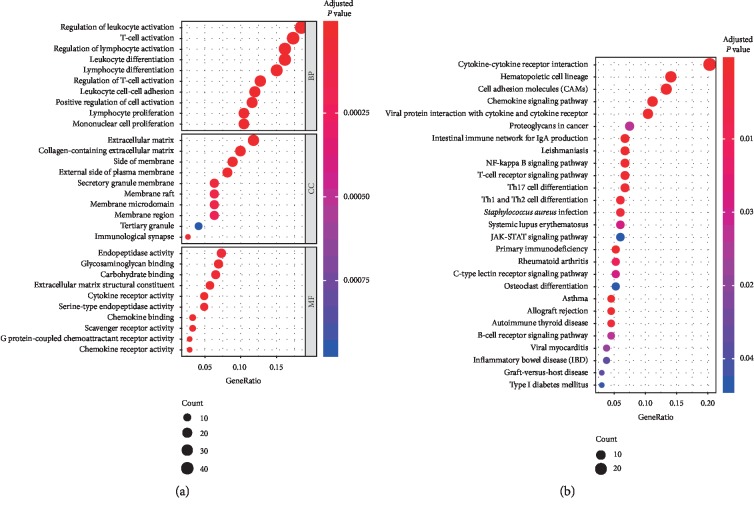
Functional enrichment analysis results of DEGs. (a) GO term enrichment results. (b) KEGG enrichment results.

Functional enrichment analysis was performed for DEGs by applying the clusterProfiler R package, which shows that these DEGs were highly correlated with immune response. GO term enrichment analysis (adjusted *P* value < 0.05; Figure 3(a)) indicated that DEGs were significantly enriched in the biological processes of immune cell differentiation and activation, cell component of extracellular matrix and membrane, molecular function of surface receptor activity, and protein binding. Moreover, the KEGG analysis (adjusted *P* value < 0.05; Figure 3(b)) suggested that most of DEG-related pathways were significantly linked to immune response.

### 3.4. Module Analysis from the PPI Network

**Figure 4:**
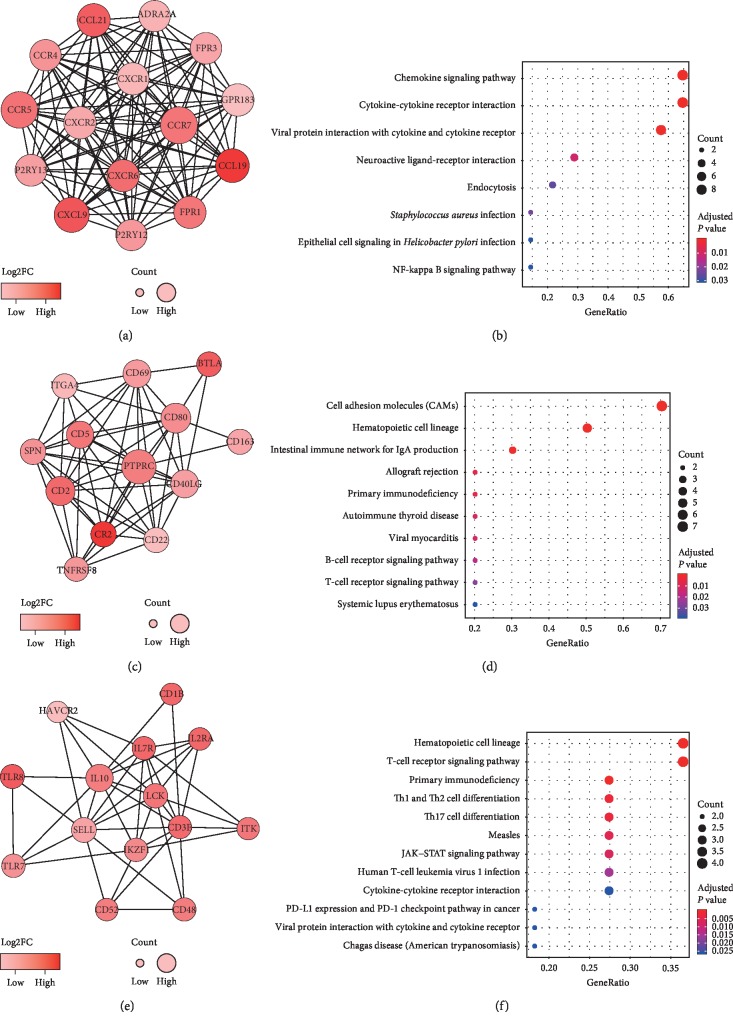
The top three significant modules from PPI network and their pathway enrichment analysis. (a) Module 1. (b) KEGG enrichment results of Module 1. (c) Module 2. (d) KEGG enrichment results of Module 2. (e) Module 3. (f) KEGG enrichment results of Module 3. In the modules, the color of a node in the PPI network reflects the log2FC value of gene expression, and the size of node indicates the count of edges with other genes.

The PPI network of DEGs was acquired by applying the online STRING tool. This network consists of nine modules, which include 74 nodes and 255 edges. The top three significant modules were selected for further analysis (Figure 4). We named these modules as Modules 1, 2, and 3, respectively. In Module 1, 15 nodes with 105 edges were formed in the network, including *ADRA2A*, *CCL19*, *CCL21*, *CCR4*, *CCR5*, *CCR7*, *CXCL9*, *CXCR1*, *CXCR2*, *CXCR6*, *FPR1*, *FPR3*, *GPR183*, *P2RY12*, and *P2RY13* (Figures 4(a) and 4(b)). Module 2 contained 54 edges involving 13 nodes: *BTLA*, *CD163*, *CD2*, *CD22*, *CD40LG*, *CD5*, *CD69*, *CD80*, *CR2*, *ITGA4*, *PTPRC*, *SPN*, and *TNFRSF8* (Figures 4(c) and 4(d)). Module 3 included 42 edges involving 14 nodes: *CD1B*, *CD3E*, *CD48*, *CD52*, *HAVCR2*, *IKZF1*, *IL10*, *IL2RA*, *IL7R*, *ITK*, *LCK*, *SELL*, *TLR7*, and *TLR8* (Figures 4(e) and 4(f)). The KEGG enrichment analysis showed that the genes in Modules 1 to 3 are mainly correlated with chemokine signaling pathway, cell adhesion molecules, and hematopoietic cell lineage.

### 3.5. Survival Analysis

**Figure 5:**
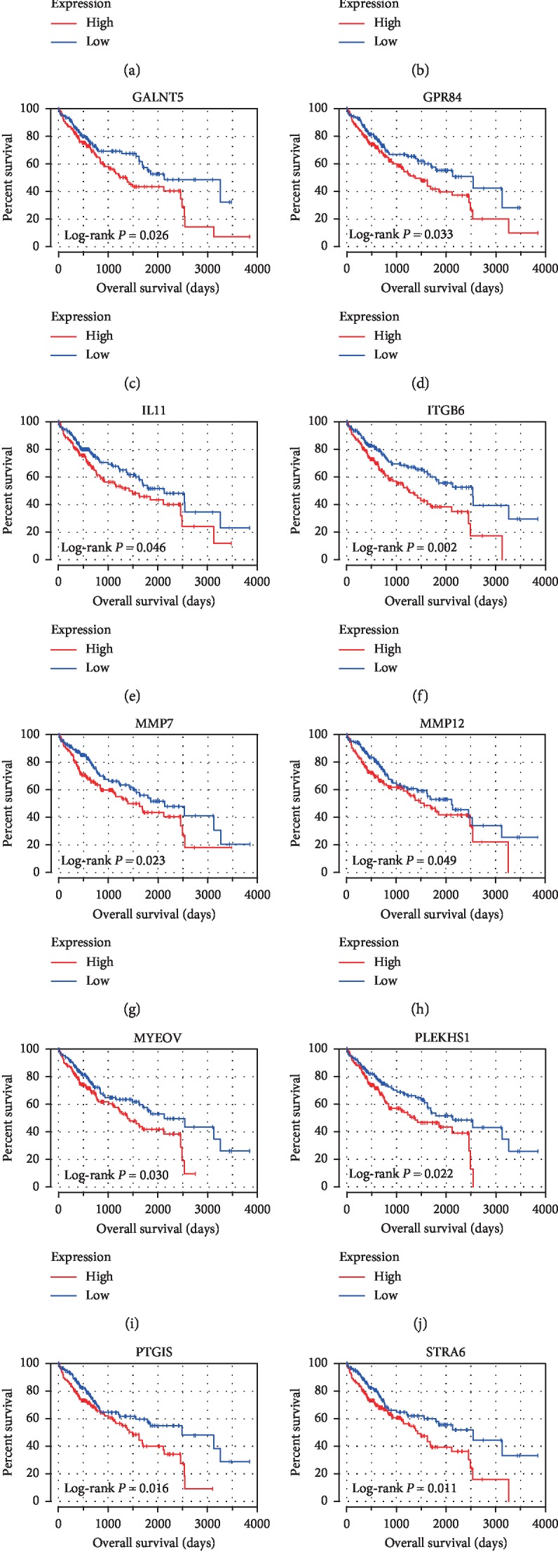
Survival analysis between DEGs and poor OS in HCC. Kaplan–Meier curves were drawn for screening DEGs with prognostic value from the comparison between high (red line) and low (blue line) gene expression groups.

To explore the underlying prognostic value of individual DEGs, the survival analysis was performed between 289 DEGs and the OS in patients with HCC from TCGA database ([Supplementary-material supplementary-material-1]). Among the 289 DEGs, a total of 12 DEGs were shown to significantly associate with poor OS (log-rank *P* < 0.05), which contain CD80, FABP3, GALNT5, GPR84, IL11, ITGB6, MMP7, MMP12, MYEOV, PLEKHS1, PTGIS, and STRA6 (Figure 5). All the 12 genes were upregulated DEGs.

## 4. Discussion

The data mining of TCGA database has been widely applied to cancer prognosis prediction, and recent studies reveal that TME plays a crucial role in HCC growth and progression [9, 25]. Therefore, we intend to identify TME-related genes that significantly affect HCC prognosis from TCGA database in this study. Particularly, these genes associate with stromal and immune components in the TME.

First, we acquired the stromal and immune scores to determine whether they were associated with the clinical characteristics and OS of HCC patients. The results show that they were indeed related to the indicators of clinical progress and prognosis, such as liver fibrosis/cirrhosis status, MVI, histological grade, and serum AFP. The multivariate Cox regression model based on stromal and immune scores showed that the risk score was significantly associated with 3-year OS, and time-dependent ROC analysis demonstrated that this prognostic risk score model performed well in 3-year OS prediction. Next, 289 DEGs were screened by comparing the high- and low-score groups of stromal and immune cells. Subsequent GO term analysis found that most of them were involved in TME, and the KEGG pathways analysis also shows that most of the DEGs were significantly associated with immune response, which is consistent with previous reports stating that the functions of stromal and immune components are interrelated in constituting TME in HCC [12, 14, 26]. Then, we constructed the PPI network to better understand the interactions of DEGs, and the top three modules show that they were all significantly related to the pathway of immunologic and inflammatory response. *CCR7*, *PTPRC* (*CD45*), and *IL10* were the most connected nodes in these modules, in which *CCR7* was a crucial molecule in the mechanism of HCC's progression and metastasis [27–29], *PTPRC* involved in the regulation of cytokine-induced signaling in malignancies [30–32], and *IL10* has been reported to increase the susceptible risk of HCC [33], decrease immunologic activity [34], and promote immune tolerance in the tumor milieu [35, 36]. Finally, survival analysis was performed to explore the potential prognostic value of 289 DEGs, and we identified 12 TME-related genes that showed significant correlation between gene expression and poor outcomes in HCC cases. Of the 12 genes, five genes (*CD80*, *IL11*, *MMP7*, *MMP12*, and *PTGIS*) have been reported to be associated with HCC's progression or significant in HCC survival prediction [37–42], indicating that our big data analysis based on ESTIMATE algorithm has prognostic values in the HCC cohort of TCGA database. The other seven genes have never been reported to correlate with HCC development and prognosis before and can be perceived as potential biomarkers for HCC.

Among the seven potential biomarkers *FABP3*, *GALNT5*, *GPR84*, *ITGB6*, *MYEOV*, *PLEKHS1*, and *STRA6*, we are particularly interested in *GPR84* and *STRA6* because they link to liver fibrosis that is a major risk factor in HCC and an independent risk factor of recurrence after hepatectomy [43, 44]. *GPR84*, a protein-coding gene of the metabolic G protein-coupled receptor family, plays a potential role in the lipid metabolism and regulation of inflammation. The latest study demonstrates that *GPR84* is involved in fibrotic pathway and *Gpr84* knockout model in mice can reduce the degree of fibrosis [45]. Moreover, targeted GPR84 treatment has been shown to be effective in liver fibrosis [46]. Therefore, *GPR84* enables the promotion of liver fibrosis and is deleterious in chronic liver disease, which may be the cause of pathogenesis and progression in HCC. Likewise, *STRA6*, as a coding gene of membrane protein involved in the metabolism of retinol, is also reported to be involved in relevant signaling of fibrosis [47] and has been shown to inhibit the effectiveness of antifibrotic treatment [48]. Interestingly, fibrosis is characterized by the excessive accumulation of extracellular matrix in damaged or inflamed tissues [49], which indicates from another aspect that *GPR84* and *STRA6* are related to TME component. The other five candidate genes are *FABP3*, *GALNT5*, *ITGB6*, *MYEOV*, and *PLEKHS1*. *FABP3* belongs to the intracellular fatty acid-binding protein family, which is thought to participate in the uptake, intracellular metabolism, and transport of long-chain fatty acids, and may be responsible for the modulation of cell growth and proliferation [50]. Recent study has suggested that *FABP3* is upregulated in hepatic steatosis in zebrafish model, and hepatic steatosis can be ameliorated by suppressing *FABP3* expression in the liver [51]. Hepatic steatosis, like the nonalcoholic fatty liver disease, is also a major risk factor for HCC. Thus, further investigation is necessary to identify the potential biological relevance between *FABP3* and HCC. *GALNT5* encodes a membrane-bound transferase in the Golgi and is reported to facilitate the proliferation and migration of colorectal and gastric cancer cells [52, 53]. *ITGB6* encodes a protein that is a member of the integrin superfamily. Members of this family are adhesion receptors that function in signaling from the extracellular matrix to the cell. A study demonstrates that *ITGB6* is expressed in malignant colonic epithelia and is associated with the progression, metastasis, and chemotherapeutic resistance of colon cancer [54]. *MYEOV* gene is localized at chromosome 11q13 that is a frequent site for chromosomal rearrangements in various carcinomas and B-cell neoplasms [55]. Previous reports suggest that the expression of *MYEOV* is enhanced in non-small-cell lung cancer and colorectal cancer and promotes cancer cell proliferation and invasion [56, 57]. For *PLEKHS1*, the proteins encoded by *PLEKHS1* participate in intracellular signaling. Meanwhile, *PLEKHS1* is able to cause noncoding mutations by regulating recurrent mutations of upstream and promoter elements, which can lead to tumorigenesis [58]. Currently, although the functional verification experiments of the seven genes in HCC have not been reported, they are more or less associated with the occurrence and development of malignancies and still need further investigation.

The studies reported in the past decade have been able to delineate the landscape of genomic alterations and gene signatures occurring in HCC growth and progression [59]. This delineation has certainly changed our perception of the disease. Meanwhile, great development has been made on the correlation of prognostic prediction with the gene expression in HCC. Many of these studies were performed through the construction of animal models, experiments of cell in vitro, and small-scale cohorts of clinical tumor samples. However, the complex interplay of HCC and the microenvironment where it is located demands a highly comprehensive analysis of large-scale cohorts. Fortunately, due to the significant progress of whole-genome sequencing technology, some high-throughput tumor databases, such as TCGA, have been developed and are publicly available for open academic communication. These platforms can provide resources for big data analysis with large-scale cohorts of HCC or other malignancies.

Compared with previous reports that focused on how the activation of tumor intrinsic gene exerts an influence on the TME, our study attaches high importance to gene signatures in TME, which in turn act on HCC's development, hence affecting patients' prognosis. Our study may provide additional data and new ideas to analyze the complex interactions between HCC and the TME where it is located. However, some limitations exist in this study which still needs to be elaborated. First the clinical information from the TCGA database is incomplete, such that detailed data about the treatment after surgery are unavailable. As a result, we were not able to perform a comprehensive survival analysis that considered other potential prognostic factors in HCC. Second, HCC cases in this study are exclusively obtained from a single cohort, which may have caused biases to our results. Third, our findings still need further validation, which may be performed through confirmatory experiments with real-time PCR and Western blot for screened genes.

## 5. Conclusions

In conclusion, we calculated the stromal and immune scores based on the ESTIMATE algorithm to identify 12 TME-related genes with poor prognosis from the HCC cohort of TCGA database. Seven of the 12 genes, namely, *FABP3*, *GALNT5*, *GPR84*, *ITGB6*, *MYEOV*, *PLEKHS1*, and *STRA6*, can be perceived as candidate genes for the prognostic prediction of HCC, which have not been previously reported for their prognostic value in HCC patients. Further study on these genes can contribute to an in-depth and comprehensive understanding of the potential correlation between TME and HCC prognosis. In addition, we anticipate that our strategy of mining TME-related genes can be widely applied to big data analysis and discover more biomarkers with prognosis value for HCC or other malignancies.

## Figures and Tables

**Table 1:** Distribution of HCC patients' characteristics and their clinical correlation with stromal and immune scores.

Variables	Count (total *n* = 374)	Stromal scores		Immune scores	
		Median	*P* value	Median	*P* value
*Age (years)*			0.488		0.868
≤60	177 (47.3%)	−661.51		416.99	
>60	193 (51.6%)	−707.93		437.03	
NA	4 (1.1%)	—		—	

*Gender*			0.940		0.326
Female	121 (32.4%)	−667.77		386.47	
Male	250 (66.8%)	−690.11		441.65	
NA	3 (0.8%)	—		—	

*Liver fibrosis/cirrhosis*			0.565		0.013^a^
No	74 (19.8%)	−713.70		243.15	
Yes	138 (36.9%)	−657.35		522.19	
NA	162 (43.3%)	—		—	

*Pathologic stage*			0.180		0.084
Stage I	171 (45.7%)	−656.40		454.29	
Stage II	86 (23.0%)	−748.34		503.16	
Stages III and IV	90 (24.1%)	−752.87		306.66	
NA	27 (7.2%)	—		—	

*Histologic grade*			0.014^a^		0.938
G1	55 (14.7%)	−385.71		307.42	
G2	177 (47.3%)	−658.31		459.21	
G3	122 (32.6%)	−771.93		424.43	
G4	12 (3.2%)	−1041.72		540.85	
NA	8 (2.2%)	—		—	

*Serum AFP*			0.013^a^		0.533
≤400 ng/mL	213 (56.9%)	−647.06		458.80	
>400 ng/mL	65 (17.4%)	−824.84		447.73	
NA	96 (25.7%)	—		—	

*Child-Pugh score*			0.350		0.207
A	217 (58.0%)	−697.06		416.70	
B and C	22 (5.9%)	−790.82		158.16	
NA	135 (36.1%)	—		—	

*MVI*			0.018^a^		0.644
No	206 (55.1%)	−590.14		424.57	
Yes	109 (29.1%)	−755.81		416.70	
NA	59 (15.8%)	—		—	

*Radical resection*			0.478		0.552
R0	324 (86.6%)	−703.78		412.96	
R1 & R2	18 (4.8%)	−527.29		467.77	
NA	32 (8.6%)	—		—	

^a^The value of *P* < 0.05 indicates statistical significance; NA: not available.

## Data Availability

The datasets analyzed during the current study are available from the corresponding author on reasonable request.

## Supplementary Materials

Supplementary Materials Table S1: the survival analysis results of the 289 DEGs.
